# SMART Stone Multidisciplinary Team (MDT) and patient care: recommendations for the adult high-risk kidney stone patient pathway

**DOI:** 10.1007/s00345-025-05602-8

**Published:** 2025-04-22

**Authors:** Bhaskar Somani, Esteban Emiliani, Thomas Knoll, Giorgia Mandrile, Gill Rumsby, Cecile Acquaviva, Naeem Bhojani, Saeed Bin Hamri, Ewa Bres-Niewada, Niall F. Davis, Daniel G. Fuster, Sander F. Garrelfs, Vineet Gauhar, Shuzo Hamamoto, Patrick Juliebø-Jones, Marta Leporati, Emmanuel Letavernier, Tatsuya Takayama, Lazaros Tzelves, Steffi Kar Kei Yuen, Pietro Manuel Ferraro

**Affiliations:** 1 SMART Stone Steering Committee, Southampton, UK; 2 Department of Urology, University Hospital Southampton, Southampton, UK; 3 Department of Urology, Fundació Puigvert, Universitat Autonoma de Barcelona, Barcelona, Spain; 4 Department of Urology, NYU Langone Health, NYU Grossman School of Medicine, New York, USA; 5 Department of Urology, University Medicine Mannheim, Mannheim, Germany; 6 Genetic Unit and Thalassemia Centre, San Luigi University Hospital, Turin, Italy; 7 University College London Hospitals NHS Foundation Trust (Retired), London, UK; 8 Service Biochimie et Biologie Moléculaire, Centre de Biologie et Pathologie Est, Unité Maladies Héréditaires du Métabolisme, CHU de Lyon, France; 9 University of Montreal Hospital Center, Université de Montréal, Montreal, QC Canada; 10 Division of Urology, King Abdulaziz Medical City, Riyadh, Saudi Arabia; 11 Department of Urology, Faculty of Medicine, Roefler Memorial Hospital in Pruszkow, Lazarski University, Warsaw, Poland; 12 Department of Urology, Beaumont Hospital, Dublin, Ireland; 13 Department of Nephrology and Hypertension, Inselspital, Bern University Hospital, University of Bern, Bern, Switzerland; 14 Department of Pediatric Nephrology, Emma Children’S Hospital, University of Amsterdam, Amsterdam, The Netherlands; 15 Department of Urology, Ng Teng Fong General Hospital, Singapore, Singapore; 16 Department of Nephro-Urology, Nagoya City University Graduate School of Medical Sciences, Nagoya, Japan; 17 Department of Urology, Haukeland University Hospital, Bergen, Norway; 18 Laboratorio di Chimica Analitica e Calcolosi Renale, SC Laboratorio analisi, Azienda Ospedaliera Ordine Mauriziano di Torino, Turin, Piemonte Italy; 19 UMR S 1155, Sorbonne Université, 75020 Paris, France; 20 Department of Urology, International University of Health and Welfare Hospital, Nasushiobara, Japan; 21 Department of Urology, Sismanogleio Hospital, National and Kapodistrian University of Athens, Athens, Greece; 22 Department of Surgery, SH Ho Urology Centre, the Chinese University of Hong Kong, Hong Kong SAR, China; 23 Section of Nephrology, Department of Medicine, Università Degli Studi di Verona, Verona, Italy; 24 Royal College of Surgeons in Ireland (RCSI), Dublin 2, Ireland

**Keywords:** Complex stone disease, Consensus, High-risk kidney stone, Multidisciplinary team, Patient care

## Abstract

**Purpose:**

The SMART Stone Multidisciplinary Team (MDT) recommendations aim to provide guidance on the role of the MDT in the early identification, referral and assessment of adult high-risk recurrent kidney stone formers to advance patient care.

**Methods:**

Recommendations were developed by the expert Steering Committee (SC) comprising of three Urologists, one Nephrologist, and two Biochemists/Geneticists from the UK, Spain, Germany, and Italy. These recommendations were voted on by invited specialists via an online survey to determine their level of agreement, from ‘strongly agree’ to ‘strongly disagree’. With an agreement threshold set at ≥ 70%, the SC reviewed the survey results, additional comments, and any areas of disagreement before finalizing the recommendations.

**Results:**

A total of 44 recommendations were developed by the SC designed to support the set-up of an ideal MDT. Thirteen core recommendations were chosen as being highest priority and were voted on by 29 invited specialists from 19 countries across Europe, Canada, East Asia, South/Southeast Asia, and the Middle East. All 13 core recommendations reached the ≥ 70% agreement threshold. The remaining 31 recommendations were voted on by those specialists who opted-in to partake in the extended questionnaire. Fifteen specialists provided their responses from 14 different countries. All 31 recommendations reached the ≥ 70% agreement threshold.

**Conclusions:**

An ideal MDT process can achieve comprehensive, high-quality, and coordinated patient care, which is especially useful for patients with complex stone diseases. A high level of agreement was reached in areas relating to the implementation of an ideal MDT in identifying high-risk stone formers.

**Graphic abstract:**

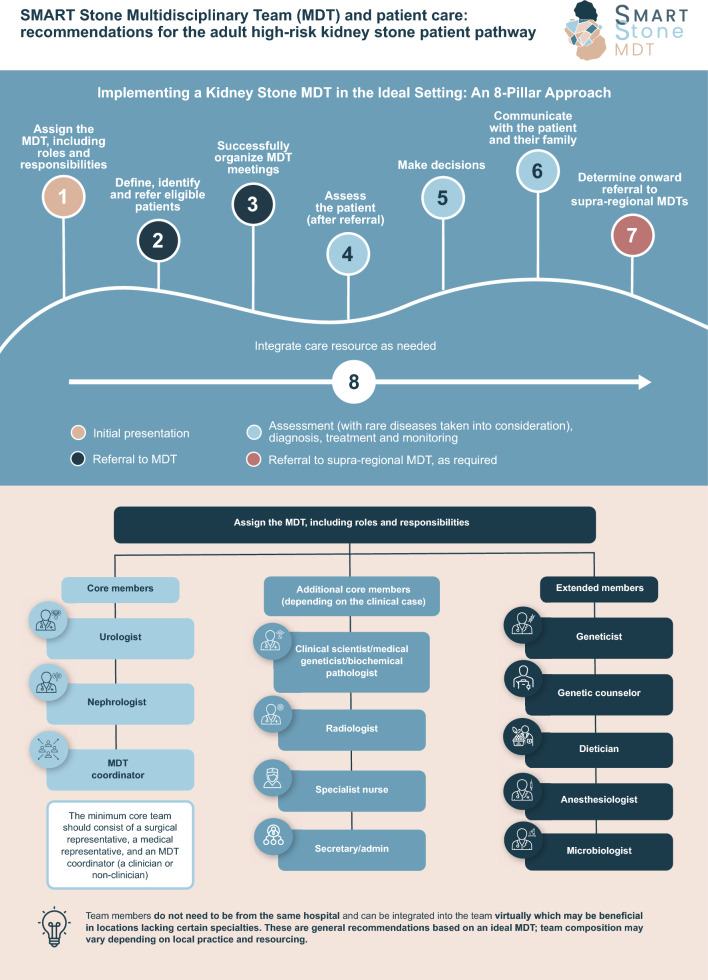

## Introduction

Multidisciplinary teams (MDTs) are made of individuals from different disciplines or stages of patient management who come together to coordinate and organize healthcare services [1]. This allows collaboration aimed to achieve accurate diagnosis and best clinical outcomes based on the clinical scenario and, if possible, patient preference. An ideal MDT process can guarantee comprehensive, high-quality patient care, which is especially beneficial in patients with complex medical backgrounds, comorbidities, or complex diseases such as recurrent stone disease [2, 3].

MDTs are established and common practice in complex disease areas, such as oncology, [1, 2, 4] and have shown benefits to patients, healthcare professionals (HCPs), organizations and healthcare systems, including adherence, reduced time to diagnosis, and better treatment outcomes [4].

With a rise in the prevalence of kidney stone formers over the last three decades, kidney stones present challenges for patients, physicians, and healthcare systems [5–7]. High-risk recurrent kidney stone formers have substantial morbidity and reduced quality of life (QoL), which increases financial and resourcing burden on healthcare systems due to multiple hospital admissions and surgical procedures [6–8].

As a multifactorial disease, a close interplay is needed between medical management, surgical management, and biochemical and genetic analysis, to name a few, thus highlighting the need for an MDT approach [5, 7, 9, 10].

Currently, best practice recommendations on how to form an MDT to support the management of adults with complex stone disease are lacking. We propose a ‘SMART’ Stone MDT to provide recommendations on the role of the MDT in early identification, referral, and assessment of adult high-risk recurrent kidney stone formers to advance patient care.

## Methodology

**Fig. 1:**
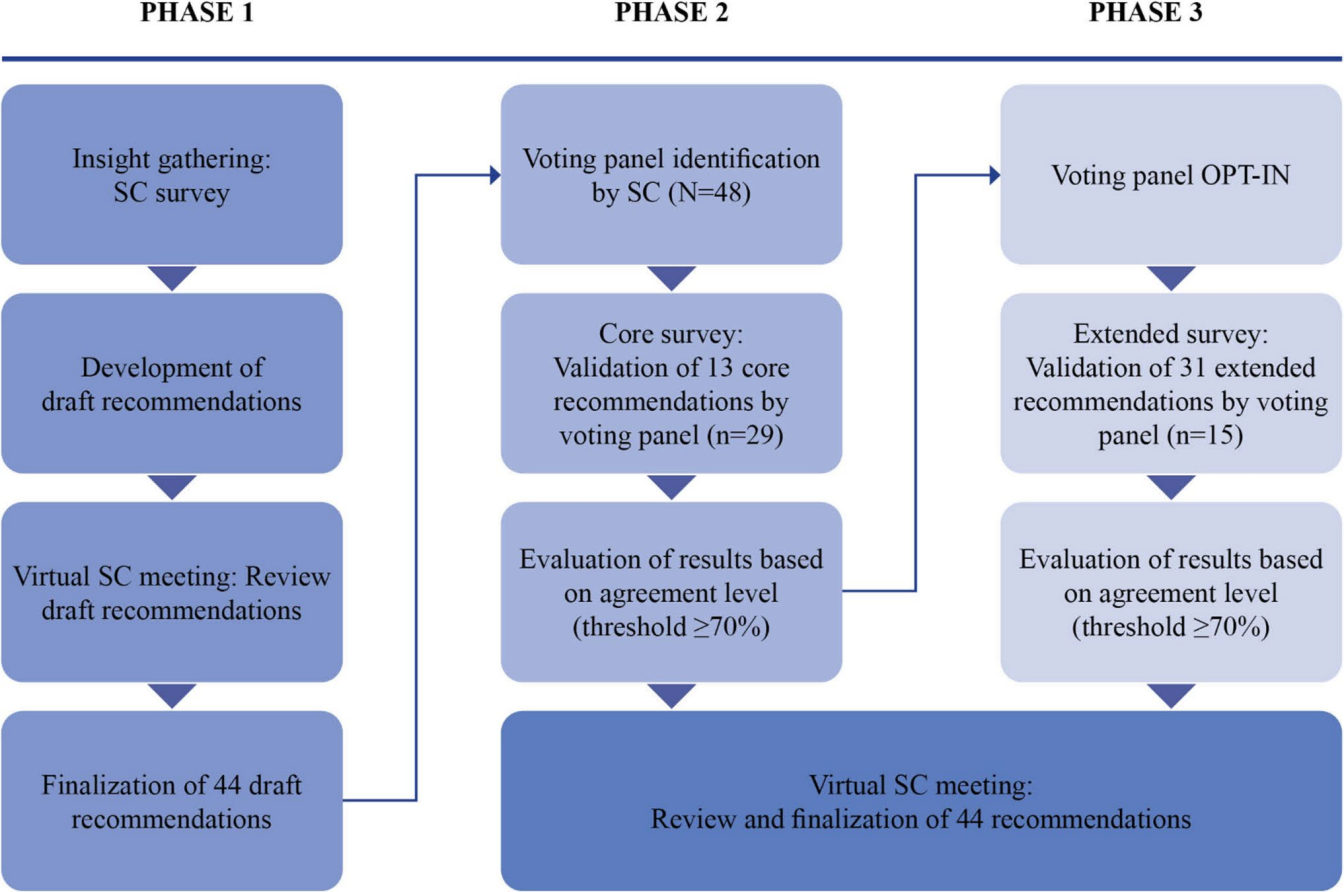
Methodology overview

The project was carried out using a three-phase process consisting of initial recommendation development, panel voting of core recommendations, and panel voting of extended recommendations before finalization (Fig. 1).

### Recommendation development

Recommendations were developed by the SMART Stone Steering Committee (SC), consisting of six experts (three Urologists, one Nephrologist, and two Biochemists/Geneticists) from the UK, Spain, Germany, and Italy. An initial questionnaire was developed to gain insights from the experts relating to best practices in MDT implementation for early identification of high-risk stone formers in the adult setting. Initial draft recommendations were agreed upon and validated during a virtual meeting ahead of voting by the extended voting panel.

### Extended voting panel

An extended voting panel of experts were recommended by the SC and invited to complete a core online questionnaire via email, consisting of the 13 highest priority recommendations in establishing an MDT in the ideal setting.

After completion of the core questionnaire, an optional invitation was extended to the voting panel to participate in an extended questionnaire comprising of additional recommendations drafted by the SC.

The panel was asked to review and rank each recommendation in the core (13 recommendations) and extended questionnaire (31 recommendations) based on their level of agreement on a five-point scale from ‘strongly agree’, to ‘strongly disagree’ (strongly agree, agree, neither agree nor disagree, disagree, and strongly disagree). Panelists also had the opportunity to supply additional comments throughout the survey, and if ‘disagree’ or ‘strongly disagree’ were chosen, panelists were asked to provide a rationale and/or suggest revisions to the recommendation. Panelists who were unsure or unconfident in choosing an adequate response based on their experience were asked to select ‘Don’t know/outside of my expertise’.

Based on prior consensus method research, consensus was defined as an agreement level (panelist selecting ‘agree’/’strongly agree’) of ≥ 70% of responders [11, 12]. Percentage agreement was calculated for each statement, and “Don’t know/outside of my expertise” responses were also included in the calculation to represent the true percentage of agreement across the panel. Results and any additional comments on areas of disagreement were discussed by the SC in a virtual meeting before the recommendations were finalized.

## Results

A total of 44 recommendations were developed by the SC, designed to support the set-up of an ideal MDT including team composition, patient identification and referral, planning and coordination, patient assessment, decision-making, communication, onward referral, and care integration. Thirteen core recommendations were chosen as the highest priority for establishing an MDT with the remaining 31 being part of the extended recommendations.

### Core recommendations

Forty-eight specialists were invited to complete the core survey, with 29 responding (22 Urologists, 5 Nephrologists and 2 Biochemists/Geneticists) from 19 different countries (Canada [n = 3], France [n = 3], Italy [n = 3], Greece [n = 2], Japan [n = 2], Saudi Arabia [n = 2], Spain [n = 2], China, Denmark, Hong Kong, Ireland, Israel, Nepal, The Netherlands, Norway, Poland, Singapore, Switzerland, and Turkey [all n = 1]).

All 13 core recommendations reached the ≥ 70% agreement threshold, with 12 recommendations reaching a ≥ 90% agreement level (Table 1). Overall, 93% (n = 27/29) of responders agreed or strongly agreed that an MDT is required to improve not only the therapeutic management but also the patient journey and to provide the best outcomes for patients with complex stones.

**Table 1:** Summary of the SMART Stone MDT core recommendations (250 words)

No	Summary of the SMART stone MDT core recommendations		Agreement level* N = 29, % (n)
Initial opinion on forming a stone MDT			
1	A stone MDT is required to improve the patient journey and provide the best outcomes for patients with complex stones		93 (27)
Section 1: Team composition *Please note, these are general recommendations; team composition may vary depending on local practice and resourcing*			
2	A core team member is defined as a critical team member for the MDT who is involved in the day- to-day management of the patient • Core team members can consist of a Urologist, Nephrologist, Biochemical Pathologist/Clinical Scientist/Medical Geneticist, Radiologist, Specialist Nurse, MDT Coordinator, and Secretary/Admin staff depending on availability and resourcing within local areas		97 (28)
3	It is recommended to have a minimum of three core team members within the MDT, this could include a Urologist (surgical representative), Nephrologist (medical representative) and an MDT coordinator (which could be a clinician, e.g., Urologist/Nephrologist, or a non-clinician, e.g., Secretary) • Additional core members can also be included, e.g., Biochemical Pathologist/Clinical Scientist/Medical Geneticist, Nurse Specialist, Radiologist, and Secretary/Admin, depending on availability and resourcing as well as the patient needs and complexity of the case		100 (29)
4	An extended team member is defined as an important team member who is not critical to the MDT or day-to-day management of the patient • Extended team members can be called upon specifically by the core MDT and can consist of a Geneticist, Genetic Counselor, Dietician, Anesthesiologist or Microbiologist to name a few. Other extended members can be included		90 (26)
Section 2: Patient identification and referral			
Recommendations for defining a high-risk stone former			
5	High-risk stone formers can be defined according to the EAU guidelines, with additional criteria that can be considered as stated below		100 (29)
	General factors associated with high-risk stone formers, as per the EAU guidelines: [10] Early onset of urolithiasis Family history of stones Recurrent stones Short time since last stone episode Brushite-containing stones (CaHPO4.2H2O) Uric acid and urate-containing stones Infection stones Solitary kidney Chronic kidney disease (CKD)	Additional criteria to be considered when assessing the risk of stone formation: Active disease Reduced estimated glomerular filtration rate (eGFR) Bilateral stones Nephrocalcinosis Unusual stone composition Cystine-containing stones Stones of genetic causes (e.g., Xanthine and 2,8-dihydroxyadenine) Known/suspected secondary stone disease Surgical or reintervention risks Younger age (e.g., < 25 years [19])	
Recommendations for referring a high-risk stone former			
6	To ensure effective use of the MDT, high-risk stone formers should be referred if they meet any of the following absolute criteria; all other patients should be considered case-by-case according to the additional criteria below *Please note that, not all criteria need to be met for referral to the MDT, and is up to the discretion of the referring physician and the MDT*		97 (28)
	Criteria for an absolute referral to the MDT: Known/suspected rare disease Infection stones Familial history of genetically caused stones (if known family history with multiple family members; if unknown, testing can be conducted by the MDT, as appropriate) Complex patients, including complications of surgery Pre-operation planning in complex stones Complex stones, including unusual stone composition Cystine-containing stones Stones of genetic causes (e.g., Xanthine and 2,8-dihydroxyadenine)	Criteria to consider a referral to the MDT: Early onset of urolithiasis/nephrocalcinosis Known/suspected secondary stone disease Disease predisposing to stone formation Solitary kidney Recurrent stones Active disease Younger age of onset Reduced eGFR Strong family history	
Recommendations for identifying a high-risk stone former			
7	Multiple evaluations/tests should be conducted for early identification of high-risk stone formers. These could be conducted ahead of MDT referral, if applicable, and are outlined below Evaluations/tests include, but are not limited to: Blood, spot, and 24 h urine^†^ biochemical analysis Urine parameters for stone screens and serum samples in stone screen Number of stone episodes Stone analysis/composition with Fourier-transform spectroscopy (FT-IR)/x-ray diffraction (XRD) of recurrent stones Imaging Patient history, including disease predisposing to stone formation, previous stone analysis, and previous imaging from any surgery Nephrocalcinosis DNA testing based on clinical history, if applicable^‡^		100 (29)
Section 3: Planning and coordination			
Recommendations for preparing for an MDT meeting			
8	An MDT meeting should be focused with clear outcomes that can be communicated to the patient. To prepare for an MDT meeting, it is recommended the following should be provided ahead of time: A list of patients for discussion A full clinical summary of the patient to be discussed, this could include frequency of stones, patient history, imaging, laboratory results, genetic results, and physical examination notes, as applicable Imaging should be reviewed ahead of the meeting Questions pertaining to the discussion should be prepared in advance, especially if relating to a complex case/imaging Clinicians will have the responsibility to present the clinical cases they see in the clinic or after/during hospitalization		100 (29)

Section 4: Patient assessment by MDT (after referral)		
Recommendations for better management of high-risk patients in relation to rare disease		
9	Suspicion of a rare disease should be raised by the MDT and, if necessary, discussed with the Geneticist as soon as possible. Genetic testing should be performed only after genetic counseling, depending on availability of Genetic Counselors in local areas. Appropriate additional biochemical tests should be requested, as required. Liaison with biochemistry laboratories is recommended as tests will be non-standard for specific rare diseases	100 (29)
Section 5: Decision-making		
Recommendations for reaching a consensus when making clinical decisions with the MDT		
10	A consensus should be reached based on a case-by-case discussion. A leading clinician that has the most experience in the main clinical challenge of the patient should lead the decision-making process and make the final decision, taking advice from all MDT members	100 (29)
Section 6: Communication		
Recommendations for discussing outcomes from the MDT with patients and their families		
11	The specialist with the most experience in the main clinical challenge of the patient and who has driven the patient pathway should be responsible for discussing outcomes from the MDT meeting with patients and their families	79 (23)
Section 7: Onward referral		
Recommendations for referral to supra-regional MDTs in relation to complex cases/rare diseases		
12	If a specific expertise is required that is not available in the local area, a broader connection via a supra-regional MDT should be identified based on the local health system organization	97 (28)
13	If a local MDT does not have the possibility of genetic testing, a web discussion should be organized, with the following points considered: Pre-test counseling can be offered by an experienced Urologist/Nephrologist, after discussion with the Geneticist Post-test counseling with the patient should be managed by web consultation with the Geneticist directly with the patient, preferably with the presence of the Urologist/Nephrologist responsible for the patient	90 (26)

*Responders selecting ‘Agree’ or ‘Strongly agree’.

^†^In some instances, 24 h urine analysis will be conducted by the MDT and not ahead of referral, this is dependent on patient presentation and local logistics/resourcing.

^‡^Depending on local resources, DNA testing, and subsequent Genetic Counseling should be provided by the MDT and not ahead of referral.

### Extended recommendations

Twenty-one specialists opted-in to receive the extended questionnaire, with 15 of those providing their responses from 14 different countries (Canada, France [n = 2], Greece, Hong Kong, Ireland, Italy, Japan [n = 2], The Netherlands, Norway, Poland, Saudi Arabia, Singapore, and Switzerland). Responders were a mixture of Urologists (n = 10/15), Nephrologists (n = 3/15) and Biochemists/Geneticists (n = 2/15). Those 15 responders were subsequently invited to be part of the authorship group due to their extensive contribution to the study.

All 31 extended recommendations reached the ≥ 70% agreement threshold, with 22 recommendations reaching a ≥ 90% agreement level (Table 2). Highest agreement was achieved for recommendations relating to communication, decision-making, onward referral, and care integration. Team composition, including roles and responsibilities, had the lowest level of agreement, although still reaching the ≥ 70% threshold.

**Table 2:** Summary of the SMART Stone MDT extended recommendations (250 words)

No	Summary of the SMART stone MDT extended recommendations	Agreement level* N = 15, % (n)
Section 1: Team composition, including roles and responsibilities		
Recommendations for the composition of an ideal MDT to identify high-risk stone formers in the adult setting *Please note, these are general recommendations; team composition may vary depending on local practice and resourcing*		
14	Core and extended team members do not need to be from the same hospital and can be integrated into the team virtually depending on resourcing and team member availability in specific hospitals	100 (15)
15	The MDT should be formalized by the core team members to ensure the team are officially recognized by the hospital to enable appropriate scheduling and reimbursement, as applicable to local areas. This could also support external communication with other specialties and centers, as required during the MDT process	93 (14)
16	Roles and responsibilities of the core team can transfer between specialties depending on resourcing within local areas	80 (12)
17	It is recommended that the specialist with the most experience relevant to the main clinical challenge of the patient should have the main responsibility of the patient	93 (14)
18	Action planning and follow-up with the patient and General Practitioner (GP, e.g., to arrange scans or clinical follow-up) should be conducted by the person that has the most contact with the patient and who has driven the process during the MDT referral. If an MDT coordinator has been defined in the core team, they can also be assigned this role	100 (15)
Recommendations for the specific roles and responsibilities of the core team members *Please note, these are general recommendations, roles and responsibilities may vary depending on local practice and resourcing*		
19	The main roles and responsibilities of the Urologist from the perspective of an ideal MDT include, but are not limited to: Leading cases relating to patients on a surgical pathway Identification of high-risk patients Providing insights on surgical and medical management strategies Aligning patient and physician expectations	87 (13)
20	The main roles and responsibilities of the Nephrologist from the perspective of an ideal MDT include, but are not limited to: Leading cases relating to patients on a medical pathway Metabolic assessment and interpretation of laboratory tests (e.g., blood, 24 h urine analysis) to establish a diagnosis and/or suspicion of secondary stone disease Medical management and follow-up Renal function monitoring and renal impairment management	80 (12)
21	The main roles and responsibilities of the MDT Coordinator from the perspective of an ideal MDT include, but are not limited to: Coordination of the MDT meetings Organization of case discussion/interface with hospital administration Listing patient and outcome summaries Communication of outcomes, if applicable	100 (15)
22	The main roles and responsibilities of the Specialist Nurse from the perspective of an ideal MDT include, but are not limited to: Patient assessment, care, education, and pathway management Providing information on how to perform metabolic investigations Coordination of tests Optimization of treatment	73 (11)
23	The main roles and responsibilities of the Biochemical Pathologist/Clinical Scientist/Medical Geneticist from the perspective of an ideal MDT include, but are not limited to: Initial interpretation of laboratory tests Metabolic assessments Providing advice on further investigation for unusual tests	93 (14)

24	The main roles and responsibilities of the Secretary/Admin from the perspective of an ideal MDT include, but are not limited to: Practical organization of case discussion Scheduling patient appointments, especially in relation to transition from the urology practice to the MDT setting Collating patients Communicating, arranging necessary follow-up and listing interventions Administration and transcription of the meeting Ensuring patients are kept informed	93 (14)
25	The main roles and responsibilities of the Radiologist from the perspective of an ideal MDT include, but are not limited to: Obtaining information from imaging studies (e.g., Hounsfield units and stone burden) Interpretation of the hardness of stones/location, unless the Urologist is familiar with these parameters	87 (13)
Recommendations for the specific roles and responsibilities of the extended team members		
*Please note, these are general recommendations, roles and responsibilities may vary depending on local practice and resourcing*		
26	The main roles and responsibilities of the Geneticist from the perspective of an ideal MDT include, but are not limited to: High-risk stone patient assessment especially with family history of genetic and rare causes Genetic analysis, if genetic disorder is suspected from stone recurrence frequency Providing the optimal genetic platform (e.g., next generation sequencing) and the range of testing (e.g., whole-genome sequencing, broader panel, or selected genes) for patients selected by the core team Interpretation of genetic results including uncertain findings (e.g., variants of uncertain significance) Family counseling In circumstances where a Geneticist is not available in the local MDT, patients can be referred to a supra-regional MDT, if available	93 (14)
27	The main roles and responsibilities of the Genetic Counselor from the perspective of an ideal MDT include, but are not limited to: Family counseling if an inherited disorder is suspected, to assess risks to other family members Evaluation of patients with genetic-related stones Genetic screening and advice to prevent future recurrence In circumstances where a Genetic Counselor is not available in the local MDT, patients can be referred to a supra-regional MDT, if available	87 (13)
28	The main roles and responsibilities of the Dietician from the perspective of an ideal MDT include, but are not limited to: Collecting information and providing tailored recommendations on dietary habits for stone and recurrence prevention, if disorder is responsive to dietary intervention Patient education, counseling, and monitoring	93 (14)
29	The main roles and responsibilities of the Anesthesiologist from the perspective of an ideal MDT include, but are not limited to: Assessment of high-risk anesthetic patients Provide advice on their suitability of surgical intervention Surgery organization when required	80 (12)
30	The main roles and responsibilities of the Microbiologist from the perspective of an ideal MDT include, but are not limited to: Assessment of high-risk patients at risk of infection complications Provide advice and help with antibiotic prophylaxis and treatment Provide advice to the MDT during difficult cases with potential infection or mixed genesis stones Urine analysis	87 (13)
Section 2: Patient identification and referral		
31	It is recommended that recurrent stones can be defined as two or more new stone events in a three-year period	73 (11)
32	Development of a valid and reliable prediction model for recurrence based on stone composition/urine chemistries is needed and would be helpful to identify high-risk stone formers earlier	100 (15)
Section 3: Planning and coordination		
Recommendation for planning a meeting schedule		
33	The following points should be considered when planning a meeting schedule: Regular, fixed meetings with specific timeslots should be scheduled at least once a month, however, ideally these should be scheduled weekly Meeting length is to be determined by the MDT core team members and should be long enough in duration to discuss a selection of patients (e.g., six cases in one hour, with 10 min minimum allocated per patient) Meeting length should be sufficient to discuss each patient succinctly and will be dependent on patient complexity, imaging requirements, number of assessments (i.e., first vs subsequent assessments), and resourcing Meetings can be in-person or virtual using the hospital’s preferred platform (e.g., Zoom), depending on team availability Teams should communicate regularly via hospital-approved appropriate channels (e.g., e-mail)	87 (13)
Section 4: Patient assessment by MDT (after referral)		
Recommendations for better management of high-risk patients in relation to rare diseases		
34	Better management of high-risk patients in relation to suspected rare diseases stems from improved identification, referral mechanisms for metabolic testing, and referral to supra-regional centers for genetic and rare disease testing, as well as referral to Center of Excellence (CoE) for patient management and provision of advanced treatment options for specific rare/ultra-rare diseases A multidisciplinary approach should be taken with shared decision-making at the center of MDT outcomes. Patient expectations must be managed throughout as additional genetic and metabolic tests can take time EAU guidelines and other rare-disease specific guidelines/recommendations should be followed, along with specific reimbursement requirements	100 (15)
35	One of the main objectives of an MDT should be to reach a diagnosis and determine appropriate subsequent treatment; considerations of rare diseases should, therefore, come naturally, provided that the right genetic tools are available Red flags/risk factors should be noted according to the EAU guidelines and other rare disease-specific guidelines/recommendations, which will indicate further investigation of rare diseases A complete assessment of all required evaluations should follow EAU guidelines and other rare disease-specific guidelines/recommendations Rare diseases should be discussed with a Geneticist after exclusion of secondary stone formations, or in cases with unexpected clinical course, based on the clinical diagnosis Provide a comprehensive treatment and management plan for identified patients with a rare disease, including potential referral to dedicated CoE who manage the specific rare disease identified	100 (15)
Section 5: Decision-making		
Recommendations for reaching a consensus when making clinical decisions with the MDT		
36	Each MDT member must agree on the proposed intervention, based on each member’s competencies. All decisions should be documented with who was present during the MDT meeting	93 (14)
37	Clinical guidelines should be at the core of decision-making (e.g., EAU and other rare disease- specific guidelines), with patient preference, health and comorbidities taken into consideration. The MDT also has scope to expand on the clinical guidelines depending on clinical experience, best practice, and local hospital guidelines	100 (15)
Recommendations for ensuring patient interests are kept at the center of decision making		
Section 6: Communication		
38	Patient wishes and preferences should be taken into consideration during MDT discussions. Active follow-up is necessary for all patients The patient should receive oral and written (via letter) clear notes on the steps and recommendations provided by the MDT; the GP should also receive a copy of any correspondence The outcome of the MDT meeting should be disclosed to the patient in a dedicated meeting with at least two members of the MDT present, ideally members most involved in the patient’s follow-up The patient and family can be seen in the outpatient clinic, and the clinician should assess their expectations and interests. Discussion can take place in a urology or nephrology practice and could include other MDT members with the most relevant expertise	93 (14)
Recommendations for discussing outcomes from the MDT with patients and their families		
39	Genetic results should be discussed with the patient by the Geneticist, if available. Shared consultation with the referring clinician (e.g., Urologist/Nephrologist) and the Geneticist would be optimal to gain perspectives of both clinical management and genetics. Genetic test results should be summarized and communicated in a simple way	93 (14)
40	Metabolite test results should be communicated in a simple way and discussed point-by-point by the MDT member with the most experience	93 (14)
Section 7: Onward referral		
Recommendations for referral to supra-regional MDTs in relation to genetic analysis		
41	Ideally, a supra-regional genetic center should be identified in the absence of genetic facilities within the local MDT. The MDT should define who the available contact is at the specialist center and what cost elements need to be considered in relation to Geneticists/Genetic Counselors	100 (15)
42	Genetic analysis should be performed and assessed correctly before referral, if available	80 (12)
Section 8: Care integration		
Recommendations for an ideal model for integrated care when thinking about an MDT approach		
43	This is a complex area that should be further discussed by the MDT in its own setting. Availability of resources should also be accounted for, with the following points to be considered: Ensure the MDT has the appropriate instruments to recognize fragile patients (both physiologically and socially) and organize support when required A Psychologist can be included as an extended member during the MDT if necessary. The patient should be referred to a Clinical Psychologist or integrative medicine consultation if needed Ensure that the social and physiological needs of the patient are considered during management. If applicable, include recommendations of patient needs to the primary care provider, such as the GP Exchange information and communicate effectively with the GP, patient, and their family	93 (14)
Recommendations for local team engagement to support patients with multiple physiological and social care needs		
44	This is a complex area that should be further discussed by the MDT in its own setting. Availability of resources should also be accounted for, with the following points to be considered: Ensure effective communication with contacts within local social care teams. A reference clinician in local teams should be named, to have a direct contact to discuss the case. Social workers should be consulted if necessary Information relating to Patient Association Groups and rare disease expertise networks should be provided to patients, if applicable. Consider patient referral to a rare-disease specific CoE for optimal management and provide patients with a reference contact, if available Exchange information and communicate effectively with the GP, patient, and their family	This is a complex area that should be further discussed by the MDT in its own setting. Availability of resources should also be accounted for, with the following points to be considered: Ensure effective communication with contacts within local social care teams. A reference clinician in local teams should be named, to have a direct contact to discuss the case. Social workers should be consulted if necessary Information relating to Patient Association Groups and rare disease expertise networks should be provided to patients, if applicable. Consider patient referral to a rare-disease specific CoE for optimal management and provide patients with a reference contact, if available Exchange information and communicate effectively with the GP, patient, and their family

*Responders selecting ‘Agree’ or ‘Strongly agree’

## Discussion

A high level of agreement was reached on areas relating to the implementation of an ideal MDT in identifying high-risk stone formers, with all 44 recommendations (core and extended) reaching the ≥ 70% agreement threshold. While the recommendations focused on the ideal situation, location-specific nuances such as healthcare setting, infrastructure, and resource availability should be considered.

### Team composition

The ideal MDT comprises a core and extended team (Table 1). As a minimum, the core team should be formed of 3 members including, a surgical representative (Urologist), a medical representative (Nephrologist), and an MDT Coordinator (a clinician or a non-clinician, e.g., Secretary); additional core members should be included depending on the clinical case (Biochemical Pathologist/Clinical Scientist/Medical Geneticist, Radiologist, Specialist Nurse, and Secretary/Admin).

Extended team members (Clinical Geneticist, Genetic Counselor, Dietician, Anesthesiologist, and Microbiologist) can be called upon specifically by the core team depending on the clinical case, needs of the patient, and specific clinical questions. Depending on resource availability, the core and extended team members do not need to be from the same hospital and can be integrated into the team virtually as needed, which may be beneficial in locations that do not have access to certain specialties.

A detailed description of roles and responsibilities of each team member can be found in Table 2. The questionnaire responses highlighted variations across different healthcare systems relating to the roles and responsibilities of different members of the MDT. For example, the role of the Urologist differs in Japan compared to European countries, and the inclusion of Specialist Nurses in an MDT is more common in the UK.

Different countries may also have different qualifications for specialists. For example, a Medical Geneticist may also be known as a Molecular Geneticist, and a Biochemical Pathologist may be known as a Biochemist in different settings. These differences should be taken into consideration when implementing local MDTs.

It is important to note that team composition, and roles and responsibilities may vary and should accommodate local practices and resources.

### Patient identification and referral

High-risk stone formers should be determined holistically considering several factors, including risk of chronic kidney disease (CKD), end-stage kidney disease (ESKD), and metabolic bone disorder (MBD) [10]. High-risk stone formers can be defined according to the European Association of Urology (EAU) guidelines, with additional criteria identified by the SC (Table 1)***,*** including recurrent stones, early onset of urolithiasis, and family history of stones to name a few.

The SC outlined the criteria to determine an absolute referral of a high-risk stone former to an MDT such as known/suspected rare disease, infection stones and familial history of genetically caused stones. These criteria were supplemented with additional information to consider a referral, such as early onset of urolithiasis/nephrocalcinosis and recurrent stones (Table 1). It was noted that not all criteria need to be met for referral to an MDT, and additional patients should be considered case-by-case.

High-risk stone formers can be identified early by conducting appropriate evaluation and testing (Table 1), which could be conducted ahead of an MDT referral, if applicable. These can include blood, spot, and 24 h urine biochemical analysis, DNA testing based on clinical history, and stone analysis/composition of recurrent stones. In some instances, 24 h urine analysis and DNA testing (and subsequent genetic counseling) should be conducted by the MDT and not ahead of referral, which is dependent on patient presentation and local logistics/resourcing.

Early diagnosis is crucial in these severe and chronic conditions to allow for appropriate management and potential prevention of progression to end-stage renal disease [13].

### Planning and coordination

Successful MDT implementation relies not only on the team, but on the organization of MDT meetings, such as infrastructure, including meeting rooms/appropriate virtual meeting platforms, and logistics, including regular meetings, preparation ahead of the meeting, and post-meeting coordination of services for the patient [14].

All MDT members should prepare in advance of an MDT meeting, which should consist of regular, fixed meetings scheduled at least once a month and ideally weekly. Ways of working between team members may vary due to local hospital guidelines. These should be taken into consideration during MDT planning and coordination.

### Patient assessment by MDT (after referral)

Rare inherited metabolic disorders such as cystinuria, primary hyperoxaluria and distal tubular acidosis are associated with kidney stone recurrence [5, 13]. Although access to Clinical Geneticists and Genetic Counselors varies across healthcare settings in different countries, access to genetic facilities should be proposed as much as possible if there is any suspicion of genetic disease. Early and correct diagnosis of genetic/metabolic disease allows for preventative treatment and reduces the risk of long-term complications such as disease progression [13, 15].

### Decision-making

Guidelines are an important part of clinical practice and decision-making, hospital guidelines should be considered as well as international guidelines and recommendations to aid decision-making. It should be determined at the outset which international guidelines are to be followed e.g., EAU or American Urological Association (AUA) [10, 16, 17].

### Communication

The specialist with the most knowledge of the patient’s clinical case and established relationship should lead communication with the patient, however as previously highlighted, roles and responsibilities can transfer between team members.

In an ideal setting, two members of the MDT would be present to discuss the outcome with the patient, however this may not be feasible in all situations depending on the patient case and resources. Some patients may benefit from shared medical appointments with multiple clinicians when providing additional options to improve patient care [18].

Genetic test results should be discussed with the patient by a Clinical Geneticist, if available; however, additional members of the team with experience of genetic diseases (e.g., Nephrologist) can also discuss genetic results as needed. Additional follow-up and education would be required for both patient and their families.

### Onward referral

Supra-regional MDTs can be identified if a specific expertise is required relating to complex cases/rare diseases; this also includes access to Clinical Geneticists via supra-regional genetic centers. Local guidelines should be consulted to assess whether web consultations are appropriate for accessing pre- and post-genetic test counseling.

### Care integration

This is a complex area that should be further discussed by the MDT in its own setting, with availability of resources accounted for. The MDT should have a scientific yet holistic approach that is individualized for the patient.

### Limitation of results

As the voting panel was selected by the SC, bias may have been introduced during the selection process, with HCPs based in academic hospitals more commonly chosen. There were no specific selection criteria when choosing panel experts, however, selection was based on SC recommendation. A small number of responses were received on both core and extended surveys; further validation with a larger sample size may reduce potential bias and provide further insight.

Specialists were invited to partake in the survey from a variety of countries to ensure voting was representative of different locations; however, despite extensive follow-up, responses were not received from specialists from the US, Austria, Argentina, Belgium, Germany, and India. Certain countries were not approached such as South America and Africa due to lack of direct contact with specialists in these areas. There is also a lack of involvement of patients and patient organizations within the recommendation development. Further research, including the impact of implementation of the MDT on the patient, is warranted.

The recommendations presented are based on the ideal setting, real-world implementation will be based on individual setting and availability of resources.

## Conclusion

An ideal MDT process can achieve comprehensive, high-quality and coordinated patient care, which is especially useful for patients with complex stone disease. A high level of agreement was reached on areas relating to the implementation of an ideal MDT in early identification, referral, and assessment of adult high-risk kidney stone formers.

A broad selection of specialists, from a wide range of countries was identified for voting to ensure expertise from a variety of countries, however a limitation is the small sample size and potential selection bias in identifying the specialists.

Additional research is needed to better understand the feasibility of MDT implementation in additional countries such as the US, South America, India, and Africa as well as the impact on patient care from the patient’s perspective.

## Data Availability

No datasets were generated or analysed during the current study.
