# Correction: SMART stone multidisciplinary team (MDT) and patient care: recommendations for the adult high-risk kidney stone patient pathway

**DOI:** 10.1007/s00345-025-06059-5

**Published:** 2025-11-04

**Authors:** Bhaskar Somani, Esteban Emiliani, Thomas Knoll, Giorgia Mandrile, Gill Rumsby, Cecile Acquaviva, Naeem Bhojani, Saeed Bin Hamri, Ewa Bres-Niewada, Niall F. Davis, Daniel G. Fuster, Sander F. Garrelfs, Vineet Gauhar, Shuzo Hamamoto, Patrick Juliebø-Jones, Marta Leporati, Emmanuel Letavernier, Tatsuya Takayama, Lazaros Tzelves, Steffi Kar Kei Yuen, Pietro Manuel Ferraro

**Affiliations:** 1 SMART Stone Steering Committee, Southampton, UK; 2 Department of Urology, University Hospital Southampton, Southampton, UK; 3 Department of Urology, Fundació Puigvert, Universitat Autonoma de Barcelona, Barcelona, Spain; 4 Department of Urology, NYU Langone Health, NYU Grossman School of Medicine, New York, USA; 5 Department of Urology, University Medicine Mannheim, Mannheim, Germany; 6 Genetic Unit and Thalassemia Centre, San Luigi University Hospital, Turin, Italy; 7 University College London Hospitals NHS Foundation Trust (Retired), London, UK; 8 Service Biochimie et Biologie Moléculaire, Centre de Biologie et Pathologie Est, Unité Maladies Héréditaires du Métabolisme, CHU de Lyon, France; 9 University of Montreal Hospital Center, Université de Montréal, Montreal, QC Canada; 10 Division of Urology, King Abdulaziz Medical City, Riyadh, Saudi Arabia; 11 Department of Urology, Faculty of Medicine, Roefler Memorial Hospital in Pruszkow, Lazarski University, Warsaw, Poland; 12 Department of Urology, Beaumont Hospital, Dublin, Ireland; 13 Department of Nephrology and Hypertension, Inselspital, Bern University Hospital, University of Bern, Bern, Switzerland; 14 Department of Pediatric Nephrology, Emma Children’s Hospital, University of Amsterdam, Amsterdam, The Netherlands; 15 Department of Urology, Ng Teng Fong General Hospital, Singapore, Singapore; 16 Department of Nephro-Urology, Nagoya City University Graduate School of Medical Sciences, Nagoya, Japan; 17 Department of Urology, Haukeland University Hospital, Bergen, Norway; 18 Laboratorio di Chimica Analitica e Calcolosi Renale, SC Laboratorio analisi, Azienda Ospedaliera Ordine Mauriziano di Torino, Turin, Piemonte Italy; 19 UMR S 1155, Sorbonne Université, 75020 Paris, France; 20 Department of Urology, International University of Health and Welfare Hospital, Nasushiobara, Japan; 21 Department of Urology, Sismanogleio Hospital, National and Kapodistrian University of Athens, Athens, Greece; 22 Department of Surgery, SH Ho Urology Centre, the Chinese University of Hong Kong, Hong Kong SAR, China; 23 Section of Nephrology, Department of Medicine, Università Degli Studi di Verona, Verona, Italy; 24 Royal College of Surgeons in Ireland (RCSI), Dublin 2, Ireland

**Correction: World Journal of Urology (2025)** 10.1007/s00345-025-05602-8

The article SMART Stone Multidisciplinary Team (MDT) and patient care: recommendations for the adult high risk kidney stone patient pathway written by Bhaskar Somani · Esteban Emiliani · Thomas Knoll · Giorgia Mandrile · Gill Rumsby· Cecile Acquaviva · Naeem Bhojani · Saeed Bin Hamri · Ewa Bres Niewada · Niall F. Davis· Daniel G. Fuster · Sander F. Garrelfs · Vineet Gauhar · Shuzo Hamamoto· Patrick Juliebø Jones· Marta Leporati · Emmanuel Letavernier· Tatsuya Takayama · Lazaros Tzelves · Steffi Kar Kei Yuen · Pietro Manuel Ferrar was originally published Online First without Open Access.

After publication, the author decided to opt for Open Choice and to make the article an Open Access publication. Therefore, the copyright of the article has been changed to © The Author(s), 2025 and the article is forthwith distributed under the terms of the Creative Commons Attribution 4.0 International License, this License permits use, duplication, adaptation, distribution and reproduction in any medium or format, as long as appropriate credit is given to the original author(s) and the source, a link is provided to the Creative Commons licence, and any changes made are indicated. Please read the full license for further details at http://creativecommons.org/license s/by/4.0/.

The original article has been updated.

